# Area Deprivation Share: A New Measure of Social Need Faced by Hospitals Serving Older Adults

**DOI:** 10.1093/geroni/igab046.3305

**Published:** 2021-12-17

**Authors:** Kellia Hansmann, Ryan Powell, Amy Kind

**Affiliations:** 1 University of Wisconsin--Madison, Madison, Wisconsin, United States; 2 Geriatrics Division, Madison, Wisconsin, United States

## Abstract

Medicare’s Hospital Readmissions Reduction Program (HRRP) places disproportionate penalties on hospitals serving populations with complex medical and social needs. Without measures to identify the social need intensity of populations cared for by these hospitals, the HRRP cannot account for these risk factors, leading to burdensome penalties that may inadvertently hinder the ability of such hospitals to care for vulnerable populations. The objective of this study is to characterize the social need intensity of US hospital acute care populations. Using the Area Deprivation Index (ADI), a validated measure that ranks neighborhood socioeconomic disadvantage based on income, employment, housing, and education factors, we determined an “Area Deprivation Share” (ADS) for hospitals with 25 or more discharges using 100% of national Medicare claims data from 2013-2014. Hospital ADS is the proportion of qualifying discharges residing in the most disadvantaged neighborhoods (ADI ≥ 80th percentile) out of all qualifying discharges during the study period. Of 4,603 hospitals, median ADS was 17% (Interquartile Range: 6% - 34%). Hospitals in the highest quintile of ADS (39% to 100%), were more frequently located in small towns or isolated rural areas (52.6%, comparted to 24.2% in lower quintiles) and served a higher percentage of Black patients (19.0%, comparted to 9.7% in lower quintiles). ADS is a potential tool to inform future Medicare policy decisions. Additional research will inform how hospitals target care processes to meet the needs of older adults with complex social needs. Further study can also explore overlapping disadvantage domains of socioeconomic status, race, and rurality.

